# Interplay between exogenous and endogenous factors in seasonal vegetation oscillations

**DOI:** 10.1038/s41598-018-36898-9

**Published:** 2019-01-23

**Authors:** Omer Tzuk, Sangeeta R. Ujjwal, Cristian Fernandez-Oto, Merav Seifan, Ehud Meron

**Affiliations:** 1 Department of Physics, Ben-Gurion University of the Negev, Beer Sheva, 84105 Israel; 2 Department of Solar Energy and Environmental Physics, SIDEER, BIDR, Ben-Gurion University of the Negev, Sede Boqer Campus, Beer Sheva, 84990 Israel; 3 Complex Systems Group, Facultad de Ingeniería y Ciencias Aplicadas, Universidad de los Andes, Av. Mon. Alvaro del Portillo, 12.455 Santiago, Chile; 4 Mitrani Department of Desert Ecology, SIDEER, BIDR, Ben-Gurion University of the Negev, Sede Boqer Campus, Beer Sheva, 84990 Israel

## Abstract

A fundamental question in ecology is whether vegetation oscillations are merely a result of periodic environmental variability, or rather driven by endogenous factors. We address this question using a mathematical model of dryland vegetation subjected to annual rainfall periodicity. We show that while spontaneous oscillations do not exist in realistic parameter ranges, resonant response to periodic precipitation is still possible due to the existence of damped oscillatory modes. Using multiple time-scale analysis, in a restricted parameter range, we find that these endogenous modes can be pumped by the exogenous precipitation forcing to form sustained oscillations. The oscillations amplitude shows a resonance peak that depends on model parameters representing species traits and mean annual precipitation. Extending the study to bistability ranges of uniform vegetation and bare soil, we investigate numerically the implications of resonant oscillations for ecosystem function. We consider trait parameters that represent species with damped oscillatory modes and species that lack such modes, and compare their behaviors. We find that the former are less resilient to droughts, suffer from larger declines in their biomass production as the precipitation amplitude is increased, and, in the presence of spatial disturbances, are likely to go through abrupt collapse to bare soil, rather than gradual, domino-like collapse.

## Introduction

A pivotal question in ecology is the nature of temporal oscillations in population densities, as this variability can affect ecosystem stability and function^1–3^. Because the impact of these oscillations may be crucial for the understanding and management of natural and semi-natural ecosystems, identifying the processes that drive oscillations has been a persistent interest in the theoretical and applied ecological disciplines^1,4,5^. Processes that drive temporal oscillations are classified into two types: endogenous density-dependent processes that regulate population growth rates^6^, and exogenous environmental processes, such as rainfall variability^7–10^. While the role of endogenous factors in inducing oscillations in animal populations is well documented^1,4^, the roles of such factors in plant oscillations have been questioned^11–13^. Along with studies that do emphasize the significance of endogenous factors^14–17^, studies based on longer time series have been less conclusive, and put forth hypotheses about mechanisms that compensate for density-dependent growth, such as plasticity of plant growth and the absence of size thresholds for reproduction^5,11^.

The dispute over the source of plant oscillations stem, in part, from the difficulty in isolating the effects of endogenous vs. exogenous factors in field experiments. That difficulty can be circumvented by studying mathematical models of plant populations. A suitable context for such studies is dryland ecosystems for which annual rainfall variability is strong and mathematical models that couple plant biomass to soil moisture are available^18^. Studies of dryland-vegetation models under conditions of constant precipitation rate, which rule out exogenous factors, have identified an oscillatory instability of a steady vegetation state to uniform oscillations, but in a parameter range considered to be unrealizable in practice^19^. Realistic parameter values, however, do not rule out oscillatory decay of disturbances around a stable steady vegetation state, which reflect endogenous biomass-water relations: water availability increases biomass growth while biomass growth decreases water availability. In this paper we argue that vegetation oscillations are likely to be a result of both endogenous and exogenous factors that act in concert: endogenous processes act to induce damped oscillatory modes and exogenous factors act to pump these modes and induce large-amplitude resonant oscillations.

We use a mathematical model of dryland vegetation in a parameter range where the steady-vegetation state is a stable focus and show by multiple time-scale analysis that the application of rainfall periodicity can result in stable vegetation oscillations, the amplitude of which depends on model parameters representing species traits and environmental conditions. We then consider parameter ranges that give rise to bistability of uniform vegetation and bare soil and study numerically the implications of resonant oscillations for ecosystem function, in terms of bioproductivity and resilience to droughts, distinguishing between trait parameters representing species that have damped oscillatory modes and species that do not have such modes.

The paper is organized as follows. We begin by introducing the vegetation model we study. We then describe the results of our studies, which include: (a) the identification of oscillatory modes, (b) mathematical analysis of large-amplitude oscillations in the presence of periodic precipitation, (c) numerical studies of the implications of resonant oscillations for biomass production and ecosystem resilience in bistability ranges of uniform vegetation and bare soil, and (d) numerical studies of spatial aspects of resilience in systems subjected to strong local disturbances. A discussion of the results of these studies concludes the paper.

## The model

We derive our model from a spatially explicit dryland vegetation model introduced by Gilad *et al*.^20,21^. The original model describes the dynamics of three areal-density variables representing above-ground vegetation biomass (*B*), soil water (*W*), and surface water (*H*) (all in units of kg/m^2^). We simplify this model by assuming a plane terrain, confined roots in the lateral directions and biomass-independent infiltration rate (infiltration rate in bare soil is as high as in vegetated soil). These conditions are met, for example, by Namibian grassland ecosystems^22^. For the sake of concreteness, we may therefore view *B* as representing the biomass of herbaceous vegetation. Under these assumptions the nonlocal root integrals in the original model can be integrated (practically assuming delta-function root kernels) and the equation for surface water *H* decouples from those of *B* and *W* and can be eliminated^18,23^. The Gilad *et al*. model reduces then to the following pair of coupled PDEs:

1a $${\partial }_{\tau }B={\rm{\Lambda }}WB(1-\frac{B}{K}){(1+EB)}^{2}-MB+{D}_{B}{\nabla }^{2}B,$$

1b $${\partial }_{\tau }W=P-\frac{N}{1+\frac{RB}{K}}W-{\rm{\Gamma }}W\,B{(1+EB)}^{2}+{D}_{W}{\nabla }^{2}W,$$

where ∂_*τ*_ denotes derivation with respect to time and ${\nabla }^{2}$ is the Laplacian in the *X*, *Y* plane. In the biomass equation Λ is the biomass growth rate coefficient, *K* is the maximal standing biomass, *E* is a measure for the root-to-shoot ratio, *M* is the mortality rate and *D*_*B*_ is the seed dispersal rate. In the water equation, *P* is the precipitation rate, *N* is the evaporation rate, Γ is the water uptake rate coefficient, *D*_*W*_ is the lateral soil-water diffusion rate, and *R* is a reference biomass value beyond which the reduction in water evaporation rate due to shading becomes significant. The dimensions of all quantities appearing in the model are given in Table 1. We note that the biomass variable and the space-time coordinates can be regarded as continuous variables, despite the discrete nature of plant populations and of reproduction events. In part, this is because of the high phenotypic plasticity of plants, and the ability of a single plant to change its viable biomass by orders of magnitude in response to environmental changes^18,24^.

**Table 1:** Dimensions of all quantities appearing in the model equations in terms of the fundamental dimensions: length (${\mathcal L}$), mass (${\mathcal M}$), and time (${\mathscr{T}}$).

Quantity	Dimension
*B*, *W*, *H*, *K*	${ {\mathcal L} }^{-2} {\mathcal M}$
*D*_*B*_, *D*_*W*_	${ {\mathcal L} }^{2}{{\mathscr{T}}}^{-1}$
*M*, *N*	${{\mathscr{T}}}^{-1}$
Λ, Γ	${ {\mathcal L} }^{2}{ {\mathcal M} }^{-1}{{\mathscr{T}}}^{-1}$
*E*	${ {\mathcal L} }^{2}{ {\mathcal M} }^{-1}$
*P*	${ {\mathcal L} }^{-2} {\mathcal M} {{\mathscr{T}}}^{-1}$
*τ*	${\mathscr{T}}$
*X*, *Y*	${\mathcal L}$
*R*	1

We will use units of meters (*m*), kilograms (*kg*) and years (*y*), respectively.

Rescaling the state variables *B*, *W* and the space and time coordinates as

2 $$b=\frac{B}{K},w=\frac{W{\rm{\Lambda }}}{M},x=X\sqrt{M/{D}_{B}},y=Y\sqrt{M/{D}_{B}},t=M\tau ,$$

we obtain the non-dimensional form of the model:

3a $${\partial }_{t}b=wb(1-b){(1+\eta b)}^{2}-b+{\nabla }^{2}b,$$

3b $${\partial }_{t}w=p-\frac{l}{1+\rho b}w-\gamma bw{(1+\eta b)}^{2}+\delta {\nabla }^{2}w,$$

where ${\nabla }^{2}$ denotes now the Laplacian in the plane of the dimensionless *x*, *y* coordinates, and the non-dimensional parameters are given by:

4 $$p=\frac{P{\rm{\Lambda }}}{{M}^{2}},l=\frac{N}{M},\gamma =\frac{{\rm{\Gamma }}K}{M},\rho =R,\eta =EK,\delta =\frac{{D}_{W}}{{D}_{B}}.$$

We analyzed Eq. (3) analytically, using multiple time-scale analysis (see the Supplementary Information) and numerically. Bifurcation diagrams were calculated using the numerical-continuation software AUTO-07P^25^. Stability properties of the steady states were deduced from eigenvalues in the case of constant precipitation, and from Floquet multipliers in the case of periodic precipitation. The model equations were integrated using either backward differentiation formulas or Fourth order Runge-Kutta scheme. For the spatial version of the model we used the finite-difference method to compute the space derivatives. To describe a concrete ecosystem these values should be supplemented by the values of the redundant dimensional parameters that have been scaled out. As an example, we may consider a grassland ecosystem using the parameters studied in ref. ^22^: *K* = 0.4 kg/m^2^, *M* = 10.5 y^−1^, ${\rm{\Lambda }}=0.9\,{{\rm{m}}}^{2}\,{({\rm{kg}}\cdot {\rm{y}})}^{-1}$, ${\rm{\Gamma }}=12.0\,{{\rm{m}}}^{2}\,{({\rm{kg}}\cdot {\rm{y}})}^{-1}$, *N* = 15.0 y^−1^. Accordingly, the values of non-dimensional parameters used in the paper, except otherwise mentioned, are: *γ* = 0.457, *l* = 1.428, and *δ* = 20.0.

In the first three subsections of the Results section we will be concerned with spatially uniform solutions and will therefore drop the spatial derivative terms. Such solutions are realizable with fairly uniform initial conditions and weak pattern-forming feedbacks to avoid spatial instabilities. In the reduced model (3) this amounts to choosing sufficiently small values of *δ* or *η*. In the last subsection, Fronts of oscillating vegetation, we consider nonuniform solutions and study the spatial version of (3).

## Results

### Damped oscillatory modes

**Figure 1:**
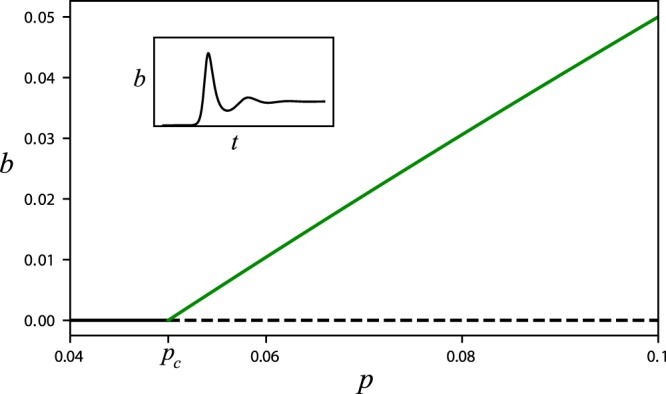
Bifurcation diagram for constant solutions of Eq. (3). Solid (dashed) lines represent stable (unstable) solutions. The bare-soil state (black line) looses stability at *p* = *p*_*c*_ = 0.05 to a steady vegetation state (green line) in a supercritical bifurcation. The inset shows the oscillatory approach to the stable vegetation state from an unstable bare-soil state, which indicates the existence of a damped oscillatory mode. Parameters: *η* = 0, *ρ* = 0, *γ* = 0.9, and *l* = 0.05.

The model equation (3) have two constant solutions, assuming constant precipitation rate *p*, as the bifurcation diagram in Fig. 1 shows. The first solution represents the bare-soil state for which *b* = 0 (black line), while the second solution represents a vegetation state with a positive biomass that increases monotonically with precipitation (green line). To facilitate the analysis of these states we consider first a simpler case for which *η* = 0 and *ρ* = 0. The bare-soil state (*b*_0_, *w*_0_) and the vegetation state (*b*_1_, *w*_1_) are then given by

5 $${b}_{0}=0,{w}_{0}=\frac{p}{l}\,{b}_{1}=\frac{p-l}{p+\gamma },{w}_{1}=\frac{p+\gamma }{l+\gamma }.$$

A linear stability analysis of the bare-soil state indicates that this state is stable up to a precipitation threshold *p*_*c*_ = *l*, and that at this threshold the vegetation state appears. A linear stability analysis of the latter state indicates the possible existence of *damped oscillatory modes*, as the numerical simulation shown in the inset of Fig. 1 demonstrates. The characteristic equation for the eigenvalues, *λ*, of the Jacobian matrix about the vegetation state is

6 $${\lambda }^{2}+2\alpha \lambda +p-l=0,\,\alpha =\frac{1}{2}[\frac{p-l}{l+\gamma }+\frac{p(l+\gamma )}{p+\gamma }],$$

and the conditions for damped oscillations are complex eigenvalues with negative real parts, or *α* > 0 and *α*^2^ − (*p* − *l*) < 0. Under these conditions the eigenvalues are

7 $$\lambda =-\,\alpha \pm i\omega ,\,{\omega }^{2}=p-l-{\alpha }^{2},$$

where the imaginary part *ω* is the frequency of the damped oscillations. Note that for the parameter values *η* = 0 and *ρ* = 0 considered here, *α* is positive throughout the existence range of the vegetation state (*p* > *p*_*c*_ = *l*)), ruling out the existence of a Hopf bifurcation to spontaneous oscillations.

### Large-amplitude oscillations

The existence of damped oscillatory modes becomes significant when we take into account the annual rainfall periodicity, as species with an internal periodicity 2*π*/*ω* that resonates with the external rainfall periodicity can respond with large-amplitude oscillations. To study resonant responses of this kind we derive an equation for the oscillations amplitude, assuming periodic precipitation of the form

8 $$p(t)={p}_{m}(1+a\,\cos \,({\omega }_{f}t)),\,0\le a\le 1,$$

where *p*_*m*_ is the mean annual precipitation, and *a* and *ω*_*f*_ are, respectively, the amplitude and frequency of the periodic precipitation. Assuming $\alpha \ll 1$, we approximate an oscillatory solution of Eq. (3) as

9 $$(\begin{array}{c}b\\ w\end{array})\approx (\begin{array}{c}{b}_{1}\\ {w}_{1}\end{array})+A(\begin{array}{c}\beta \\ 1\end{array})\,\exp (i{\omega }_{f}t)+c.c.,$$

where *A* is the oscillation amplitude. Using multiple time-scale analysis, as described in the Supplementary Information, we find that *β* = −*iω*/*γ* and obtain the amplitude equation

10 $$\dot{A}=-\,i\nu A+\frac{{p}_{m}a}{4}-\frac{1}{2}\,(\frac{{\omega }^{2}}{\gamma }+{p}_{m})\,A,$$

where, *ν* is the detuning parameter defined as *ν* = *ω*_*f*_ − *ω*. Note that the amplitude equation is derived to linear order as the oscillatory modes are damped and higher-order contributions are not needed to saturate the exponential growth of unstable modes.

Equation (10) has a stable steady state solution, *A*_0_, the modulus of which satisfies:

11 $$|{A}_{0}{|}^{2}=\frac{{p}_{m}^{2}{a}^{2}}{4\,{(\frac{{\omega }^{2}}{\gamma }+{p}_{m})}^{2}+16{\nu }^{2}}.$$

**Figure 2:**
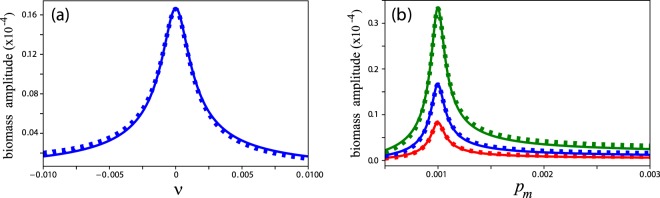
Resonant response of a system with damped oscillatory modes to periodic precipitation. Shown are the amplitude of biomass oscillations as obtained from the analytical solution (11) (solid lines) and from direct integration of Eq. (3) (squares). (**a**) The biomass oscillation amplitude as a function of the detuning parameter *ν*. (**b**) The oscillation amplitude as a function of the mean annual precipitation *p*_*m*_ for increasing values of the precipitation amplitude *a*. The parameter values were chosen to be in the validity range of the amplitude equation (10) and are given by *l* = 0.0001, *p*_*m*_ = 0.001, and *a* = 0.001 for panel (a) and *a* = 0.0005 (red), *a* = 0.001 (blue), *a* = 0.002 (green) for panel (b).

According to Eq. (9), this solution describes sustained oscillations at the forcing frequency *ω*_*f*_, i.e. a resonant response. However, the maximal amplitude of these resonant oscillations is attained only at exact resonance *ω* = *ω*_*f*_, or *ν* = 0; away from that resonance the amplitude tails off in a Lorenzian-like manner, as Fig. 2a shows. This behavior is typical to damped oscillatory systems subjected to additive periodic forcing. Since the internal frequency depends on *p*, as Eq. (7) implies, the oscillation amplitude attains a maximum also at a particular precipitation value, *p*_*max*_, as Fig. 2b shows for several forcing amplitudes. This result suggests that species with different internal frequencies show maximal oscillation amplitudes at different dimensional precipitation rates. To illustrate the significance of this point let us consider two plant species that make different tradeoffs in their investments in growth (Λ) vs. their investments in defense mechanisms against droughts (*M*), e.g. the first species has a higher growth rate than the second, Λ_1_ > Λ_2_, but also higher mortality rate, *M*_1_ > *M*_2_, such that ${{\rm{\Lambda }}}_{1}/{M}_{1}={{\rm{\Lambda }}}_{2}/{M}_{2}\equiv r$. The dimensional precipitation rates, *P*_1_ and *P*_2_, where the two species attain their maximal oscillation amplitudes are ${P}_{i}={p}_{max}{M}_{i}^{2}\,{/{\rm{\Lambda }}}_{i}={p}_{max}r{M}_{i}$ (*i* = 1, 2), and since *M*_1_ > *M*_2_ we find that *P*_1_ > *P*_2_. In other words, the fast growing species shows a maximal oscillation amplitude at a precipitation rate higher than that of the slow growing species.

The amplitude equation (10) has been derived assuming *ρ* = *η* = 0 and $\alpha \ll 1$, and, as Fig. 2 indicates, the quantitative agreement with direct simulations of Eq. (3) is very good. However, this choice of parameters requires the precipitation and evaporation rates (*p* and *l*) to be unrealistically small. In the following section we complement this analysis with numerical studies of Eq. (3) for non-zero *ρ* and *η*, relaxing the requirement of small *p* and *l*.

### Resonant oscillations and ecosystem function

**Figure 3:**
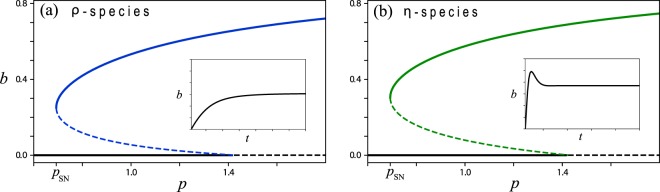
Subcritical instabilities of bare soil induced by biomass-water feedbacks. (**a**) Bifurcation diagram for a “*ρ*-species” (*ρ* = 10.1, *η* = 0) for which the shading feedback is dominant. (**b**) Bifurcation diagram for an “*η*-species” (*ρ* = 0, *η* = 3.4) for which the root-shoot feedback is dominant. The bifurcation diagrams were calculated from Eq. (3) with constant precipitation. While the *η*-species has damped oscillatory modes, as the inset in panel (b) shows, the *ρ*-species has no oscillatory modes (inset in panel (a)). The dynamics shown in the insets were calculated with *p* = 1.

The instability of bare soil to steady uniform vegetation, as the precipitation rate exceeds a threshold value, can be subcritical when the dominating feedback between biomass and water becomes strong enough to stabilize the vegetation state below the bare-soil instability^21,26,27^. The model equation (3) capture two feedbacks of this kind. The first is associated with reduced water-loss by evaporation as plants grow; while seedlings may not survive low rainfall because of high evaporation, grown plants reduce the evaporation rate by shading, and benefit from an increasing water content as they grow. This feedback is quantified by the parameter *ρ* and will be referred to as the “shading feedback”. The second feedback is associated with water uptake by plant roots; while the roots of seedlings are too small to capture enough water for seedling growth, the roots of grown plants can be large enough to sustain growth, provided the root-to-shoot ratios of these plants are sufficiently high. This feedback is quantified by the parameter *η* and will be referred to as the “root-shoot feedback”. Non-zero values of either *ρ* or *η* can indeed lead to subcritical instabilities of bare soil as Fig. 3 shows.

**Figure 4:**
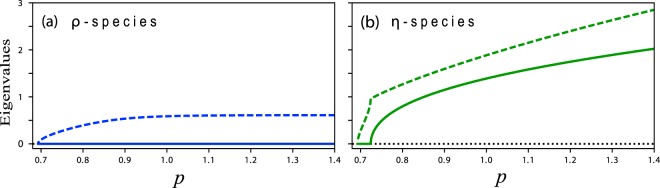
Oscillatory modes. Shown are absolute values of the imaginary parts (solid lines) and real parts (dashed lines) of the eigenvalues about steady vegetation as functions of (constant) *p*, for a *ρ*-species (**a**) and for an *η*-species (**b**). While the eigenvalues for a *ρ*-species are real valued, ruling out oscillatory modes, those of an *η*-species are complex valued, indicating the existence of damped oscillatory modes. Note that the imaginary eigenvalue part of an *η*-species, and thus its internal frequency, increases monotonically with *p*.

Associated with subcritical instabilities are hysteresis loops and loss of resilience; a productive vegetation state may undergo a sudden collapse to unproductive bare soil, as a result of a prolonged drought, and may not recover when the drought is over^18,28,29^. How do oscillatory modes affect the resilience of ecosystems to droughts? To address this question we first study how do the shading and the root-shoot feedbacks (controlled by *ρ* and *η*, respectively) affect the existence of oscillatory modes (assuming constant *p*). When both parameters are zero, oscillatory modes could be found for unrealistically small values of the evaporation rate *l* (see inset in Fig. 1). For larger values of *l* oscillatory modes can only be realized with strong root-shoot feedback ($\eta \ne 0$) as Fig. 4 shows; the shading feedback alone does not induce oscillatory modes. To discern between the effects of the two feedbacks it will be convenient to consider two hypothetical species, an “*η*-species” for which $\eta \ne 0$ and *ρ* = 0, and a “*ρ*-species” for which $\rho \ne 0$ and *η* = 0. The former shows damped oscillations (inset in Fig. 3b), while the latter does not (inset in Fig. 3a). Throughout the paper we choose the specific values *ρ* = 10.1 for a *ρ*-species and *η* = 3.4 for an *η*-species.

**Figure 5:**
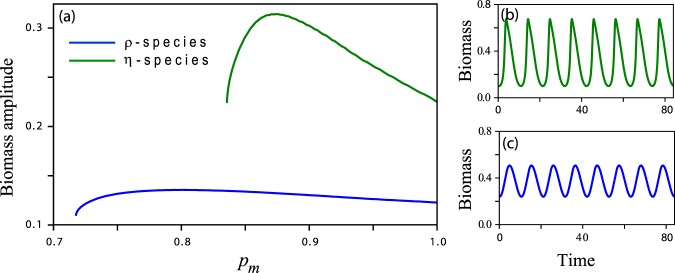
Resonant responses of *ρ*-species and *η*-species to periodic precipitation. (**a**) Biomass-oscillation amplitudes of a *ρ* species (blue line) and of an *η*-species (green line) as functions of the mean annual precipitation *p*_*m*_, obtained by numerical integration of Eq. (3) with periodic precipitation. (**b**,**c**) The biomass oscillations in time at precipitation values that correspond to the amplitude peaks in panel (a). While an *η*-species shows a pronounced high peak at a particular precipitation value at which its internal frequency *ω* resonates with the annual precipitation frequency *ω*_*f*_, no pronounced peak is found for the *ρ*-species, which does not have oscillatory modes. The calculations were made with precipitation amplitude *a* = 0.5. The non-dimensional periods of the oscillations shown in panels (b and c) correspond to 1 year for *M* = 10.5 y^−1^.

**Figure 6:**
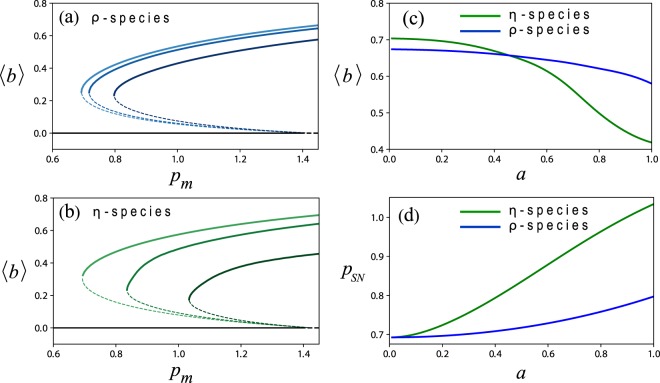
The functioning of *ρ*-species and *η*-species under periodic precipitation of increasing amplitudes. Shown are bifurcation diagrams for a *ρ*-species (**a**) and for an *η*-species (**b**) at increasing amplitudes of the periodic precipitation, where the horizontal axis represents the mean annual precipitation *p*_*m*_. The light blue and green shades in panels (a and b) denote steady-vegetation solutions obtained for constant precipitation, *a* = 0, while the darker and yet darker shades correspond to periodic precipitation with amplitudes *a* = 0.5 and *a* = 1, respectively. As the precipitation amplitude increases the biomass annual average decreases (panel (c)), and the threshold *p*_*SN*_ at which the vegetation state disappears in a saddle-node bifurcation increases (panel (d)).

The existence of oscillatory modes in the *η*-species and their absence in the *ρ*-species suggest a stronger resonant response of the former to seasonal (intra-annual) rainfall variability. One manifestation of this difference between the two species is shown in Fig. 5. While the amplitudes of resonant oscillations of both species show a peak at a particular precipitation value, as the amplitude-equation analysis in the previous section predicts, the peak of the *η*-species is much more pronounced as compared with that of the *ρ*-species. The appearance of an amplitude peak can be attributed to two factors. The first is the monotonic dependence of the internal frequency (i.e. the imaginary part of the eigenvalues about the vegetation state) on *p* (see Fig. 4b), and the existence of a particular precipitation value for which the internal frequency resonates with the external annual rainfall frequency. This factor applies to the *η*-species, which has oscillatory modes, but not to the *ρ*-species. The second factor is the decrease of the eigenvalue’s real part (in absolute value), and thus of the degree of damping, at low precipitation. This factor, which acts to increase the amplitude of oscillations, applies to both species. The stronger amplitude peak of the *η*-species is attributed in part to the resonance between the internal and the external frequencies that occurs at a particular precipitation value, a behavior that does not apply to the *ρ*-species. Another manifestation of the difference between the two species becomes apparent by comparing the bifurcation diagrams shown in Fig. 6a,b for the two species at increasing amplitudes *a* of the periodic precipitation. Two results can be inferred from these diagrams. The first is that while the mean annual biomass 〈*b*〉 of both species decreases as the precipitation amplitude *a* increases, the biomass decrease of the *η* species is significantly sharper (see Fig. 6c), suggesting lower ecosystem functioning of the *η* species in terms of biomass production. The second result is the decreasing range of existence of the vegetation state as the amplitude *a* is increased. This decrease is much sharper for the *η* species, as Fig. 6d indicates, suggesting lower ecosystem functioning in terms of poorer resilience of the *η* species to precipitation downshifts or droughts. We note that the particular values of *ρ* for the *ρ*-species and of *η* for the *η*-species were chosen so that the saddle-node bifurcation points, *p*_*SN*_, for the two species approximately coincide at *a* = 0, as Fig. 6d indicates.

### Fronts of oscillating vegetation

So far we focused on spatially uniform dynamics, which apply to situations where pattern-forming feedbacks are weak and initial conditions are restricted to be fairly uniform. In this section we still assume that the pattern-forming feedbacks are weak enough to enable bistability of uniform vegetation and bare soil, but allow for strongly nonuniform initial conditions, which in practice may result from local disturbances, such as grazing, clear-cutting, or fires. In bistability ranges of bare soil and uniform vegetation (Fig. 6a,b) such disturbances can induce local transitions to bare soil and the formation of fronts that separate vegetation and bare-soil domains. The dynamics of these fronts determine the long-term behavior of the ecosystem in question^29–31^. In the following we study the effect of periodic precipitation on front dynamics distinguishing between *ρ*-species and *η*-species.

**Figure 7:**
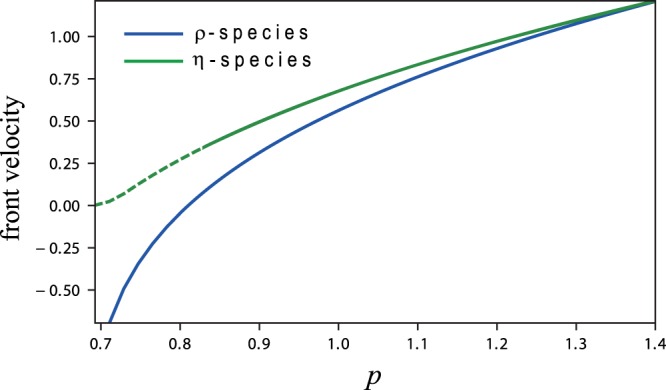
Front propagation at decreasing values of constant precipitation. Shown are velocity-precipitation relations for fronts between uniform vegetation and bare soil (solid lines) and between vegetation pattern and bare soil (dashed line). Positive front velocity corresponds to the expansion of vegetation domains into bare soil. While a front of the *ρ*-species can reverse its direction of propagation along the precipitation gradient, a front of the *η*-species retains its direction of propagation.

It is instructive to consider first the dynamics of fronts under conditions of constant precipitation. Figure 7 shows the dependence of the front velocity on the precipitation rate for *ρ*-species and *η*-species, and uncovers an essential difference: while a front of the *ρ*-species can reverse its direction of propagation as the precipitation rate is sufficiently decreased, a front of the *η*-species can slow down but cannot propagate in the opposite direction. In fact, for sufficiently low precipitation the root-shoot feedback destabilizes the uniform-vegetation state of the *η*-species into a periodic pattern (dashed green line in Fig. 7). However, that pattern keeps expanding into the bare-soil state, even though at velocities that become diminishingly low^30^. These different front-propagation properties of *ρ* and *η* species can be understood by considering the difference between the feedbacks associated with them, the shading feedback in the case of a *ρ*-species and the root-shoot feedback in the case of an *η*-species. While the shading feedback increases water availability by reducing evaporation and thus facilitates vegetation growth, the root-shoot feedback decreases water availability through competition for water and thus worsen the growth conditions. As a result, the growth conditions in a front of a *ρ*-species are worse than those within the vegetation matrix because of lower vegetation density and faster evaporation, whereas the growth conditions in a front of an *η*-species are better than those in the matrix because of reduced competition. Thus, when the precipitation rate is high and water availability in the front zone is abundant, both species expand into bare soil, but when the precipitation rate is sufficiently low, the two species behave differently. The worsen conditions in the front of a *ρ* species can lead to local mortality and to a reversal in the direction of front propagation, while the better conditions in the front of an *η* species guarantee the vegetation viability there, as long as the matrix remains viable, and reduced precipitation can only result in slowing down the expansion of vegetation into bare soil.

**Figure 8:**
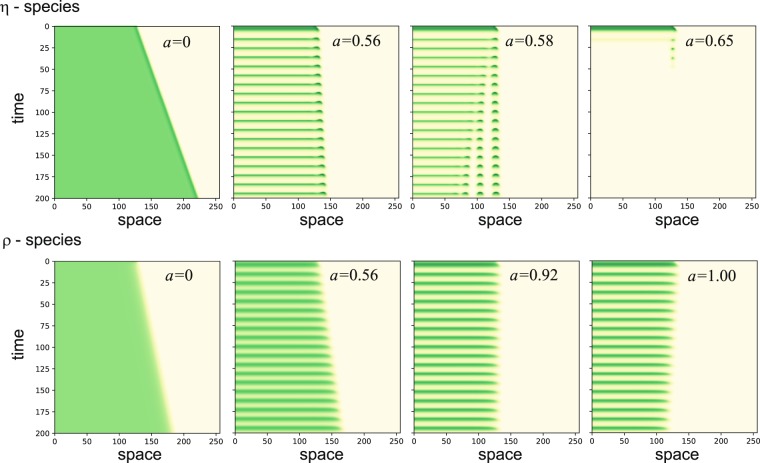
Front propagation at increasing amplitudes of the periodic precipitation. Shown are space-time plots for *η*-species (top row) and *ρ*-species (bottom row) following an initial condition representing adjacent vegetation and bare-soil domains of equal size. While fronts of the *ρ*-species can reverse their direction of propagation at high amplitudes, fronts of the *η* species can at most come to a stop. While the uniform-vegetation domain of a *ρ*-species persists at high amplitudes, that of an *η*-species destabilizes into a spatial pattern, which at yet higher amplitudes collapses to bare soil.

Another outcome of Fig. 7 is the nonlinear convex form of the velocity-precipitation relations^31^. The nonlinear form becomes significant when considering periodic precipitation, as precipitation upshifts and downshifts of equal size lead to velocity changes of different size. The convex form of these relations implies slowing down of front propagation with increasing precipitation amplitudes, as the space-time plots in Fig. 8 show. In the case of a *ρ*-species the slowing down is weak but persistent up to the maximal precipitation amplitude value *a* = 1, and involves a reversal in the direction of front propagation. In the case of an *η*-species the slowing down is stronger, but as the propagating front comes to a stop a spatial pattern develops behind the front. This oscillating pattern persists for a small range of precipitation amplitudes, but at higher amplitudes (*a* ≈ 0.65) collapses to bare soil. Note that the slowing down of a front of an *η*-species (as *a* is increased) is faster, compared to a front of a *ρ*-species, despite the fact the velocity-precipitation range of an *η*-species is less convex than that of a *ρ* species. This can be understood in terms of the resonant response of the *η* species to the precipitation periodicity. Such a response involves large amplitudes of biomass oscillations and nearly vanishing vegetation biomass during the dry season. As a result, front propagation is limited to the wet season only, resulting in a lower average velocity. The collapse of the *η*-species to bare soil at high precipitation amplitudes, unlike the *ρ*-species, is also a result of its resonant response (see Fig. 6).

These results provide new insights into desertification transitions, i.e. transitions from productive vegetation states to bare soil. As the precipitation amplitude, *a*, is increased, keeping the mean annual precipitation, *p*_*m*_, unchanged, a recovery front of the *ρ*-species (vegetation grows into bare soil) turns into a desertification front (vegetation recedes from bare soil), and *gradual desertification* takes place^29^. By contrast, a recovery front of the *η*-species never turns into a desertification front. Instead, a nearly stationary front between uniform vegetation oscillations and bare soil turns into a stationary front between oscillating vegetation pattern and bare soil, before *abrupt desertification* to bare soil takes place.

## Discussion

We present here model studies of dryland vegetation that shed new light on the much debated question whether vegetation oscillations are driven by endogenous or exogenous factors. We find that although spontaneous oscillations do not exist in physically realizable conditions, damped oscillatory modes can exist. We further find that these modes can be pumped by the annual rainfall periodicity to create large-amplitude vegetation oscillations in narrow precipitation ranges (Figs 2 and 5). This resonant response reflects a combination of endogenous factors – the oscillatory modes, and exogenous factors – the periodic precipitation. According to our model studies, the existence of oscillatory modes depends on the dominant biomass-water feedback at work; plant species with dominant root-shoot feedback (*η*-species) show damped oscillatory modes, while species with dominant shading feedback (*ρ*-species) do not show oscillatory modes. The latter follow the precipitation variability, but lack the resonant amplification that the former have. They therefore represent vegetation oscillations purely driven by exogenous factors.

The difference between the root-shoot feedback and the shading feedback (see Resonant oscillations and ecosystem function section), in terms of existence and absence of damped oscillatory modes, may be understood as follows. In the root-shoot feedback, vegetation growth acts to deplete the water resource, which feeds back negatively on further vegetation growth and thus favors oscillatory approach to steady vegetation. In the shading feedback, vegetation growth acts to reduce water loss by evaporation, which feeds back positively on further growth and favors a monotonic approach to steady vegetation. Phrased differently, the feedback that involves competition, the root-shoot feedback, gives rise to oscillatory modes, while the feedback associated with facilitation, the shading feedback, gives rise to monotonic modes. These insights may shed new light on empirical studies of damped oscillations^5,32^, which so far are inconclusive.

The appearance of an amplitude peak along the precipitation gradient is a consequence of the precipitation dependence of the intrinsic frequency associated with the damped oscillations. Since this frequency also depends on species traits, amplitude peaks for different species will generally appear at different mean annual precipitation values. This prediction implies that dominant species in a given precipitation range may become less competitive in different precipitation ranges, and be displaced by other species, assuming the precipitation is fairly periodic. Stochastic precipitation should not be expected to change significantly the results derived here for a single species, because that species will mainly respond to the precipitation frequency component that resonates with its internal frequency. In the case of a community of species, rainfall stochasticity may result in increased species diversity, as different species can resonate with different frequency components and, as a result, become more competitive. The consideration of damped oscillatory modes may therefore be of crucial importance for the understanding of community structure and dynamics in variable environments.

The distinction between vegetation oscillations driven by endogenous vs. exogenous factors is significant for assessing the resilience of dryland ecosystems to droughts. Ecosystems with damped oscillatory modes that resonate with the periodic precipitation, show high oscillation amplitudes (Fig. 5), which make them increasingly susceptible to droughts as the precipitation amplitude increases. This conclusion becomes apparent by looking at the saddle-node bifurcations points (*p*_*SN*_) of the productive vegetation-oscillations state (Fig. 6b) and their big shifts to higher precipitation values as the precipitation amplitude *a* increases (Fig. 6d). This is in sharp contrast to ecosystems that lack oscillatory modes and show low oscillation amplitudes and small shifts of the saddle-node bifurcation. Such ecosystems are less affected by increased precipitation amplitudes (Fig. 6a,c,d).

In disturbed ecosystems, the resilience to droughts is also affected by the dynamics of small bare-soil domains formed by local disturbances. Such domains may shrink and close up, or expand, depending on the propagation direction of fronts that separate vegetation and bare-soil domains (more complicated forms of front dynamics are possible, but are not considered here^31,33–36^). When these fronts are recovery fronts, describing the expansion of vegetation domains into bare soil, initial bare-soil domains close up and the ecosystem is resilient. When these fronts are desertification fronts, describing the expansion of bare soil into vegetation domains, gradual desertification takes place^29^. The front velocity is affected by the precipitation parameters, i.e. the mean annual precipitation, *p*_*m*_, and the amplitude of the periodic precipitation, *a* (Figs 7 and 8), and also by the dominating biomass-water feedback. Species with dominating shading feedback (*ρ*-species) show a transition from recovery fronts to desertification fronts as the mean annual precipitation decreases or as the amplitude of the periodic precipitation increases. By contrast, species with dominating root-shoot feedback (*η*-species) always show recovery fronts and cannot go through gradual desertification. These species can also reduce water stress by spatial patterning and, thereby, increase their resilience to droughts^23^. Yet, *η*-species are less resilient than *ρ*-species, because of their resonant response to the periodic precipitation. As Fig. 8 shows, they go through abrupt desertification (global collapse to bare soil) already at moderate precipitation amplitudes, unlike the slow gradual desertification that *ρ*-species go through, even at high precipitation amplitudes.

The results reported here are based on studies of a single vegetation model, that is, Eq. (1). However, we expect them to apply to additional vegetation models that share the same bifurcation structure and contain at least two state variables to allow for oscillatory modes. More specifically, the additional models should be capable of showing a bistability precipitation range of bare soil and uniform vegetation that terminates in a saddle-node bifurcation (*p* = *p*_*SN*_) at which the uniform vegetation state disappears (see Fig. 3). As we have already explained (see the section: Resonant oscillations and ecosystem function), bistability of this kind can be obtained either with a strong shading feedback or a strong root-shoot feedback or both. The Gilad *et al*. model and its other derivatives^23^ capture both feedbacks, while the simpler Klausmeier^19^ and Klausmeier-Gray-Scott models^37^ capture the root-shoot feedback (in the sense that the water uptake rate is proportional to the above-ground biomass). We therefore expect our results, such as early collapse to bare soil and resonant amplification of biomass oscillations, to apply to these models too. The vegetation model introduced by Rietkerk *et al*.^38^ does not capture any of these feedbacks, but slight modifications of this model (e.g. making the evaporation rate biomass dependent) can lift this limitation. In that case, we expect our results to be applicable to this model too. The Lefever-Lejeune model^26^ consists of one state variable only and, therefore, will not produce any result associated with damped oscillatory modes.

We conclude with a remark that generalizes the two hypothetical species we introduced. The terms *η*-species and *ρ*-species refer to particular parameters that control specific feedbacks in the model equation (3), the root-shoot and the shading feedbacks, and determine whether damped oscillatory modes exist or are absent. More generally, they represent two generic forms of bistable living systems consisting of functioning and dysfunctioning states. The first form corresponds to a functioning state that has complex eigenvalues and is capable of showing large-amplitude resonant oscillations in the presence of external forcing, while the second form corresponds to a functioning state that has real eigenvalues and responds to the forcing by low-amplitude resonant oscillations. The results reported here may therefore be applicable to other biological contexts of bistability that share these basic properties.

## Supplementary information

Supplementary Information
