# Supplementary material for: Interplay between exogenous and endogenous factors in seasonal vegetation oscillations
Source: Sci Rep. 2019 Jan 23;9:354. doi: 10.1038/s41598-018-36898-9 (PMC6344492; doi:10.1038/s41598-018-36898-9)
Supplement: Supplementary file 1 — Supplementary Information [file 41598_2018_36898_MOESM1_ESM.pdf]

Interplay between exogenous and endogenous factors in seasonal vegetation oscillations

Omer Tzuk[1,*], Sangeeta R. Ujjwal[2], Cristian Fernandez-Oto[2,3], Merav Seifan[4], and Ehud Meron[1,2]

[1]Department of Physics, Ben-Gurion University of the Negev, Beer Sheva, 84105, Israel
[2]Department of Solar Energy and Environmental Physics, SIDEER, BIDR, Ben-Gurion University of the Negev, Sede Boqer Campus 84990, Israel
[3]Complex Systems Group, Facultad de Ingeniería y Ciencias Aplicadas, Universidad de los Andes, Av. Mon. Alvaro del Portillo 12.455, Santiago, Chile
[4]Mitrani Department of Desert Ecology, SIDEER, BIDR, Ben-Gurion University of the Negev, Sede Boqer Campus 84990, Israel
[*]omertz@post.bgu.ac.il

Supplementary Information

Supplementary Methods M1 - Derivation of the amplitude equation

We briefly sketch here the derivation of Eq. (10) in the main text for the oscillation amplitude A introduced in Eq. (9) in the text. This is the amplitude of an oscillatory solution of Eqs. (3) with periodic precipitation given by Eq. (8). To facilitate the derivation we consider the simpler case where $\eta = \rho = 0$, and rewrite Eqs. (9) in terms of the deviations $v = b - b_1$ and $u = w - w_1$ from the steady vegetation state (b_1, w_1) (see Eq. (5):

$$\partial_t v = \frac{l-p}{l+\gamma}v + \frac{(p-l)(\gamma+l)}{(p+\gamma)^2}u - \frac{p-\gamma-2l}{p+\gamma}uv - \frac{p+\gamma}{l+\gamma}v^2 - v^2u\,, \tag{Aa}$$

$$\partial_t u = \frac{-p(l+\gamma)}{p+\gamma}u - \frac{\gamma(p-\gamma)}{l+\gamma}v - \gamma uv\,. \tag{Ab}$$

Equations (A) do not have an oscillatory instability of the steady vegetation state, but such an instability can be approached by assuming the absolute value of the negative real part of the eigenvalue (7) to be small, that is $\alpha \ll 1$ (see also Eq. (6). To this end we introduce an auxiliary small parameter ε and assume the following scaling relations: $l = \varepsilon^2 l_0$, $\gamma = \gamma_0$, $\omega = \varepsilon\omega_0$ and $p(t) = p_0\varepsilon^2\left(1+\varepsilon^2 a_0\cos\left[(\omega_0\varepsilon + \nu_0\varepsilon^2)t\right]\right)$, where ν_0 is the frequency detuning defined as $\nu_0\varepsilon^2 = \nu = \omega_f - \omega$. In these relations all quantities with subscript zero are of order unity. Under these assumptions the real eigenvalue part, or the damping, scales like ε^2 while the imaginary eigenvalue part, or the oscillation frequency, scales like ε.

Rewriting Eqs. (Aa) and (Ab) in terms of ε and retaining terms up to order ε^4 we obtain

$$\partial_t v = \frac{\varepsilon^2\omega_0^2(\varepsilon^2 l_0 - \gamma_0)}{\gamma_0^2}v + \frac{\varepsilon^2\omega_0^2(\gamma_0 - 2\varepsilon^2 p_0 + \varepsilon^2 l_0)}{\gamma_0^2}u + \frac{(\gamma_0 + 2\varepsilon^2 l_0)}{\gamma_0}uv + \frac{(-\varepsilon^2\omega_0^2 + \gamma_0)}{\gamma_0}v^2 - v^2u \tag{Ba}$$

$$\partial_t u = \frac{(\varepsilon^4 p_0 l_0 - \varepsilon^2\gamma_0\omega_0^2 - \gamma_0^2)}{\gamma_0}v - \frac{\varepsilon^2 p_0(\varepsilon^2\omega_0^2 - \gamma_0)}{\gamma_0}u - \gamma_0 uv + \varepsilon^4 p_0 a_0\cos\left[(\omega_0\varepsilon + \nu_0\varepsilon^2)t\right]. \tag{Bb}$$

We now expand solutions of Eqs. (B) in powers of ε as

$$v = \varepsilon^3 v_3 + \varepsilon^4 v_4 + \ldots, \tag{Ca}$$
$$u = \varepsilon^2 u_2 + \varepsilon^3 u_3 + \ldots, \tag{Cb}$$

and introduce a slow time variable $T = \varepsilon^2 t$. Inserting the expansion (C) into Eqs. (B) and transforming the time derivative as $\partial_t \to \partial_t + \varepsilon^2 \partial_T$, we obtain at the leading order equation:

$$\mathbf{L_0} \begin{bmatrix} v_3 \\ u_2 \end{bmatrix} = 0, \qquad \mathbf{L_0} = \begin{bmatrix} -\partial_t & \frac{\varepsilon^2 \omega_0^2}{\gamma_0} \\ -\gamma_0 & -\partial_t \end{bmatrix}. \tag{D}$$

The solution of this linear equation can be written as

$$\begin{bmatrix} v_3 \\ u_2 \end{bmatrix} = C(T) \begin{bmatrix} -\frac{i\omega_0}{\gamma_0} \\ 1 \end{bmatrix} e^{i\varepsilon\omega_0 t} + c.c., \tag{E}$$

where C is a slowly varying amplitude that depends on the slow time variable T. At the next order we obtain:

$$\mathbf{L_0} \begin{bmatrix} v_4 \\ u_3 \end{bmatrix} = \begin{bmatrix} -i\varepsilon^5 \left(\frac{\omega_0^3}{\gamma_0^2} C + \frac{\omega_0}{\gamma_0} \partial_T C \right) \\ \varepsilon^4 \left(p_0 C + \partial_T C + \frac{p_0 a_0}{2} e^{i\nu_0 T} \right) \end{bmatrix} e^{i\omega_0 t_0 \varepsilon} + \begin{bmatrix} i\varepsilon^5 \left(\frac{\omega_0}{\gamma_0} C^2 \right) \\ 0 \end{bmatrix} e^{2i\omega_0 t_0 \varepsilon} + c.c.. \tag{F}$$

Since the linear operator L_0 is singular, i.e. it has eigenvectors (E) with zero eigenvalues as Eq. (D) implies, a solvability condition should be applied[1]. To this end we consider the space of periodic vector functions with period $2\pi/\varepsilon\omega_0$ on which the following inner product between two vector functions, $f = (f_1, f_2)$ and $g = (g_1, g_2)$, is defined:

$$< g|f >= \sum_i < g_i|f_i >, \qquad < g_i|f_i >= \int_0^{2\pi/\varepsilon\omega_0} g_i^*(t) f_i(t) dt.$$

The solvability condition amounts to the requirement that the right hand side of Eq. (F) is orthogonal to the zero eigenvectors $\mathbf{e}_\pm$ of the adjoint operator $L_0^\dagger$, given by:

$$\mathbf{e}_\pm = \begin{bmatrix} 1 \\ \mp i\varepsilon \frac{\omega_0}{\gamma_0} \end{bmatrix} e^{\mp i\varepsilon\omega_0 t}, \quad \text{where} \quad \mathbf{L_0^\dagger e_\pm} = 0, \qquad \mathbf{L_0^\dagger} = \begin{bmatrix} \partial_t & -\gamma_0 \\ \frac{\varepsilon^2 \omega_0^2}{\gamma_0} & \partial_t \end{bmatrix}. \tag{G}$$

Applying this condition, we obtain

$$\partial_T C = -\frac{1}{2} \left(\frac{\omega_0^2}{\gamma_0} + p_0 \right) C + \frac{p_0 a_0}{4} e^{i\nu_0 T}. \tag{H}$$

Defining the amplitude $A = \varepsilon^2 C e^{-i\nu_0 T}$, and going back to the original unscaled quantities we obtain the amplitude equation (10) in the text.

References

1. Meron, E. *Nonlinear Physics of Ecosystems* (CRC Press, Taylor & Francis Group, 2015).
